# Traditional methods *v.* new technologies – dilemmas for dietary assessment in large-scale nutrition surveys and studies: a report following an international panel discussion at the 9th International Conference on Diet and Activity Methods (ICDAM9), Brisbane, 3 September 2015

**DOI:** 10.1017/jns.2018.4

**Published:** 2018-04-02

**Authors:** B. Amoutzopoulos, T. Steer, C. Roberts, J. E. Cade, C. J. Boushey, C. E. Collins, E. Trolle, E. J. de Boer, N. Ziauddeen, C. van Rossum, E. Buurma, D. Coyle, P. Page

**Affiliations:** 1 MRC Elsie Widdowson Laboratory, Cambridge, UK; 2 NatCen Social Research, London, UK; 3 The Nutritional Epidemiology Group, The University of Leeds, Leeds, UK; 4 Cancer Epidemiology Programme, The University of Hawaii Cancer Center, Honolulu, Hawaii, USA; 5 School of Health Sciences, Faculty of Health and Medicine and Priority Research Centre in Physical Activity and Nutrition, University of Newcastle, Callaghan, NSW, Australia; 6 Division of Risk Assessment and Nutrition, National Food Institute, Technical University of Denmark (DTU Food), Kgs. Lyngby, Denmark; 7 Centre for Nutrition, Prevention and Health Services, National Institute for Public Health and the Environment (RIVM), Bilthoven, The Netherlands

**Keywords:** Dietary assessment technologies, Nutrition surveys, Mobile applications, Web-based tools, AES, Australian Eating Survey, AHS, Australian Health Survey, AMPM, Automated Multiple-Pass Method, ASA24, Automated Self-Administered 24-hour Recall, DNFCS, Dutch National Food Consumption Survey, ICDAM9, 9th International Conference on Diet and Activity Methods, mFR, mobile food record, MRC, Medical Research Council, NDNS, National Diet and Nutrition Survey, NHANES, National Health and Nutrition Examination Survey, RP, Rolling Programme, WebDASC, Web-based Dietary Assessment Software for Children

## Abstract

The aim of the present paper is to summarise current and future applications of dietary assessment technologies in nutrition surveys in developed countries. It includes the discussion of key points and highlights of subsequent developments from a panel discussion to address strengths and weaknesses of traditional dietary assessment methods (food records, FFQ, 24 h recalls, diet history with interviewer-assisted data collection) *v.* new technology-based dietary assessment methods (web-based and mobile device applications). The panel discussion ‘Traditional methods *v.* new technologies: dilemmas for dietary assessment in population surveys’, was held at the 9th International Conference on Diet and Activity Methods (ICDAM9), Brisbane, September 2015. Despite respondent and researcher burden, traditional methods have been most commonly used in nutrition surveys. However, dietary assessment technologies offer potential advantages including faster data processing and better data quality. This is a fast-moving field and there is evidence of increasing demand for the use of new technologies amongst the general public and researchers. There is a need for research and investment to support efforts being made to facilitate the inclusion of new technologies for rapid, accurate and representative data.

Recent technological advances offer opportunities to enhance the way in which dietary information is captured in nutrition surveys with potential to make a positive impact on cost, researcher and respondent burden, data quality, efficiency of data collection, coding of dietary intake and processing data, response rates and objectivity of assessment measures^(^^1^^)^. New technologies include web-based tools and mobile device applications used to automate collection of food consumption data (self-completed or interviewer-assisted), coding of foods and portion sizes, and to facilitate accurate self-completion, including visual cues for measurement guides and embedded standards. Table 1 gives an overview of these technologies and current level of use^(^^2^^–^^17^^)^.

**Table 1.** Different levels of application of new technology to dietary assessment from minimal to more extensive

Technology application	Examples
Researcher-assisted dietary assessment tools administered via computerised systems: Researcher/interviewer guides the participant during the recording of the dietary assessment	Computer-assisted personal interviews/computer-assisted telephone interview (CAPI/CATI)^(^^2^^)^ US Department of Agriculture's Automated Multiple-Pass Method (AMPM), an interviewer-assisted computer-assisted method^(^^3^^)^ DISHES software (Diet Interview Software for Health Examination Studies)^(^^4^^)^ GloboDiet software (previously EPIC-Soft^(^^5^^,^^6^^)^), a computerised standardised international 24 h dietary recall
Self-administered dietary assessment tools administered via web-based systems/mobile applications: Individual completes the diary assessment tool by themselves	Web-based Dietary Assessment Software for Children (WebDASC), self-administered web-based 24 h dietary assessment tool^(^^7^^,^^8^^)^ Automated Self-Administered 24-hour Recall (ASA24)^(^^9^^)^ The online Food4Me FFQ^(^^10^^)^ Australian Eating Survey (AES), online FFQ^(^^11^^)^ Oxford WebQ, web-based method for assessment of previous 24 h dietary intakes^(^^12^^)^ Myfood24, online 24 h dietary assessment tool^(^^13^^)^ INTAKE24, online 24 h dietary recall system^(^^14^^)^
Automated image-based dietary assessment tools administered via mobile applications/servers: Images captured by users ‘before’ and/or ‘after’ eating occasions which are, in some tools, sent to the server for automatic image analysis and results sent back to the mobile device for confirmation and review	Mobile food record (mFR), an integrated dietary assessment system supporting automatic image analysis^(^^15^^)^ Food Record App (FRapp), mobile phone food record application^(^^16^^)^ Nutricam Dietary Assessment Method (NuDAM), mobile phone image-based dietary assessment method^(^^17^^)^

Whilst there are quite a few examples of intervention studies using technology to measure dietary intake^(^^18^^)^, there are fewer examples from large-scale epidemiological studies and nutrition surveys^(^^19^^)^. To date, the latter large-scale studies and surveys have continued to use traditional methods of dietary data collection, namely tools such as food records, FFQ, 24 h recalls and diet history with interviewer-assisted data collection whether on paper or via computer, and using food models and images for quantification, and in the case of diet diaries, possible inclusion of household scales for weighing foods consumed by the respondent. Examples include the following.

The UK National Diet and Nutrition Survey Rolling Programme (NDNS RP) has used estimated 4-d diet diaries recorded using a paper form^(^^20^^)^, the Danish National Survey of Diet and Physical Activity (4–75 years) (DANSDA, 2011–2013) used a paper-based 7-d food diary^(^^21^^)^, and the Dutch National Food Consumption Survey (DNFCS)^(^^22^^)^ and the French Nutrition and Health Survey (ENNS, 2006−2007)^(^^23^^)^ used repeated 24 h recalls assisted by interviewer. More recently 24 h recalls and FFQ have been administered via computerised systems for reasons of automatic coding, reduced error and cost savings^(^^24^^)^. Recent examples of these systems are the online Food4Me FFQ^(^^10^^)^, the DISHES software^(^^4^^)^, the GloboDiet software (previously EPIC-Soft^(^^5^^,^^6^^)^) and the 24 h computer-assisted personal interviews/computer-assisted telephone interview (CAPI/CATI)^(^^2^^)^. Sweden used a web-based food record (*RiksmatenFlex*) for dietary assessment in the adult (2010–2011)^(^^25^^)^, and adolescent (2016–2017)^(^^26^^)^ nutrition surveys. However, even with these automated systems, nutrition surveillance work is challenging and misreporting of dietary intake remains problematic as in other studies where dietary assessment is undertaken. Under-reporting of food intake is one of the fundamental misreporting issues impeding the capture of accurate habitual dietary intake data. The prevalence of under-reporting in nutrition surveys was previously reported as ranging from 18 to 53 % of the whole sample^(^^27^^)^. In nutrition surveys, the success of the method relies equally on the mode of delivery to facilitate effective participation and ensuring collection of the highest-quality data which minimises measurement error. Since the 1970s, there have been efforts to develop methods to reduce error in self-assessed dietary intake data. Technological advances have emerged as a promising way forward to continue identifying and mitigating measurement errors in dietary assessment in population studies^(^^19^^,^^28^^)^. Achieving effective participation is an another issue in nutrition surveys as there is evidence that people are generally less inclined to take part in research, which could impact on response rates and not necessarily related to the use of specific tools^(^^29^^,^^30^^)^.

Another challenge in nutrition surveys is to obtain a dietary assessment system that is efficiently integrated with an accurate, comprehensive and relevant food composition dataset, maintained according to country-specific protocols. Technology offers a wide range of feasible options for dietary assessment, such as using barcodes for automated food matching^(^^31^^,^^32^^)^. Brand-level foods have been added to generic items in the searchable database of myfood24, an online 24 h dietary assessment tool^(^^13^^)^, allowing for greatly expanded food choice (about 45 000 foods)^(^^33^^)^. In the USA, the National Health and Nutrition Examination Survey (NHANES) is also benefiting from a branded food products database^(^^34^^)^. This approach potentially supports easier selection of foods actually consumed by participants instead of trying to find the closest match with smaller, generic databases. However, it also runs risk of overwhelming the participant with extensive lists of products, and may lead to bias through the participants selecting the first option they come to which resembles their food, rather than reviewing all options to select the best match.

The objective of the present paper is to report the dietary assessment methods used in current nutrition surveys across developed countries and to explore the relative merits and potential of implementing new technologies to capture dietary intake data.

## Methods

A panel symposium entitled ‘Traditional methods *v.* new technologies: dilemmas for dietary assessment in population surveys’ at the 9th International Conference on Diet and Activity Methods (ICDAM9) on 3 September 2015 in Brisbane brought together international researchers involved in dietary assessment aspects of nutrition surveys from Australia, Denmark, the Netherlands, the UK and the USA.

The aim of the symposium was to discuss the advantages, challenges and opportunities for using new technologies in national nutrition surveys and large population studies. The symposium began with presentations from the panel on their experiences of using new and emerging technologies, including future plans and challenges for implementation into nutrition survey settings. This was followed by a panel discussion focused on opportunities, practicalities and next steps.

The present paper summarises key points of the ICDAM9 2015 symposium presentations and discussion, and provides an update on recent developments and the experiences in different country surveys.

## Results

### Challenges and advantages of new technologies in nutrition surveys: examples from different countries

#### Australia

Professor Clare Collins (University of Newcastle, Australia) presented on the recent Australian Health Survey 2011–13 (AHS)^(^^35^^)^. This survey collected two 24 h recalls using the computer-assisted system called the Automated Multiple-Pass Method (AMPM)^(^^35^^)^ adapted to the Australian Food, Supplement and Nutrient Database^(^^36^^)^. The first 24 h recall was obtained using CAPI and the second, in a population subsample, conducted by interviewers using CATI^(^^35^^)^.

While a robust method, the 24 h recall is associated with considerable respondent and interviewer burden in the AHS. The AMPM could take 45–60 min to complete. Furthermore, the 24 h recall method relied on memory; therefore errors in reporting foods or portion sizes could occur. Under-reporting was identified as a major issue in the 2011–13 AHS, with rates higher than the 1995 Survey. Under-reporting of energy was determined as about 17 % in males and about 21 % in females using the cut-offs for energy intake:BMR^(^^37^^)^.

Professor Collins presented an online suite of validated semi-quantitative FFQ, the Australian Eating Surveys (AES) which have been developed to enable more frequent data collection on public health nutrition and might have wider applicability for large studies^(^^11^^,^^38^^,^^39^^)^. The AES is a 120-item FFQ with fifteen supplementary questions including food and sedentary behaviours, and supplement use^(^^11^^)^, aiming to capture the usual dietary intakes of children, adolescents and adults over the previous 6 months. A key advantage of using the AES for population surveys would be reduced administrative and participant burden (15–20 min to complete online), with data available immediately. The online AES data can generate a personalised dietary feedback in real time^(^^11^^)^ which could potentially be an important incentive to encourage participation. Use of such online FFQ, to complement data collected from other methods like the AMPM, could be tested to examine the impact on response rates. However, this approach does have some limitations, such as its ability to deliver quantitative detailed data at the individual level, and in isolation of other strategies might not fulfil all nutrition monitoring requirements. The advantages and challenges in the AES are presented in Table 2, including the potential implications and approaches of implementing new technologies.

**Table 2.** Advantages and challenges of current dietary assessment methods used in nutrition surveys and the potential use of new technologies as presented at the 9th International Conference on Diet and Activity Methods (ICDAM9) Panel 2015

Survey, country	Dietary assessment method	Advantages of the current method	Challenges of the current method	Potential benefits of new technologies
Australian Health Survey 2011–13 (AHS), Australia	Two 24 h recalls including interviewer-assisted data collection via computerised Automated Multiple-Pass Method (AMPM)	Provides detailed intake data (additional information collected including supplement use and dietary behaviours); ease of application among those with low literacy and older adults	Expensive and time consuming; possible recall bias; trained interviewer required; respondent burden	Online FFQ – the Australian Eating Survey (AES): Lower respondent burden (as takes 15 min to complete); relatively inexpensive; does not require trained interviewers; fewer data processing requirements: provides personalised dietary feedback in real time; possibility to monitor response rates
Danish National Survey of Diet and Physical Activity (DANSDA), Denmark 4–75 years old: 2003–2008, 2011–2013, and 6–36 months old: 2006–2007	Paper 7-d estimated pre-coded diary with open-answer possibilities and pre-coded answer options for the most commonly eaten foods and dishes in the Danish diet	Ease of application among those with low technology usage and older adults; ease of coding process due to precoding	Expensive and time consuming; trained interviewer required; respondent burden; slow data processing and reporting timeline; generic-level food intake information	Web-based 7-d food record (6–36 months old, 2014–2015): Lower respondent burden; richer foods and portion size options; partly automated food coding; advanced food identification and search features; automated prompts through web; personal text messages
The Dutch National Food Consumption Survey (DNFCS), the Netherlands	Two 24 h recalls using computerised GloboDiet with trained interviewers	Provides detailed intake data (provides additional information including supplement use)	Expensive and time consuming; possible recall bias; trained interviewer required; respondent burden; slow data processing and reporting timeline; difficulty to keep up-to-date food composition database; low respondent motivation (method perceived as ‘old-fashioned’)	Barcoding technology (in combination with GloboDiet): Advanced linkage of food consumption and food composition data using artificial intelligence techniques Use of mobile applications instead of GloboDiet to achieve more advanced level of food identification
National Diet and Nutrition Survey Rolling Programme (NDNS RP), UK	Paper 4-d estimated food diary	Provides detailed intake data (provides additional information on supplement use); ease of application among those with low technology usage and older adults; provides contextual eating information	Expensive and time consuming; trained interviewer required; large respondent burden (high motivation required); slow data processing and reporting timeline	Web-based 24 h recall: Cost-effective and time saving; less respondent burden; possible increase in response rates of those with high motivation for automated methods (e.g. teenagers); ease of data processing and reporting

#### Denmark

The most recent Danish Nutrition Survey, DANSDA 2011–13, used a 7-d pre-coded food diary with open answer possibilities and pre-coded answer options for the most commonly eaten foods in the Danish diet^(^^21^^)^. Associate Professor Ellen Trolle from the Technical University of Denmark presented an overview of their recent work to explore and develop more time- and cost-effective dietary assessment methods.

Dr Trolle described a self-administered web-based 24 h dietary assessment tool called WebDASC (Web-based Dietary Assessment Software for Children), which was developed and tested among children aged 8 to 11 years for 7 d recording^(^^7^^,^^8^^)^. The tool has the potential to be applied to other age groups. A modified version of WebDASC was recently used in the 2014–15 Danish nutrition survey among infants and young children (6–36 months). Compared with the more generic foods available in the paper diary, using WebDASC allowed a more varied selection of foods and of portion sizes. Professor Trolle noted that although WebDASC was well accepted among the study population and reduced data management time, there was still an associated staff cost (e.g. personal assistance for instructions, telephone hotline and reminders) and highlighted that misreporting remained a risk as in paper-based methods. A recent study in Norway^(^^40^^)^, using a modified version of WebDASC, showed that 36–37 % of children and adolescents of the study population were under-reporters and only 2 to 4 % were over-reporters. In a comparison study, the energy intake of school children aged 8 to 11 years estimated by WebDASC was compared with the total energy expenditure derived from accelerometers. It was identified that approximately 20 % of children using WebDASC were over-reporters and 20 % were under-reporters using confidence limits of agreement between energy intake and total energy expenditure^(^^7^^)^. However, the 2014–15 survey using WebDASC showed more under-reporting among toddlers compared with the paper-based method used in the 2006–07 survey^(^^41^^)^. The figures suggest misreporting varies according to various factors like age^(^^42^^)^ in addition to the administration of tools. In light of these challenges for nutrition surveys, suggestions for the future were to optimise dietary data collection through WebDASC by using technological advances such as three-dimensional images for portion size estimation and speech search for spelling competences of children^(^^7^^)^. In 2017 a study was initiated to evaluate the implementation of the 2 × 24 h recall method (combined with a FFQ) as recommended by the European Food Safety Authority^(^^2^^)^ or the web-based 7-d food diary by using doubly labelled water techniques and nutrition biomarkers to assess the intake of fruits and vegetables and fatty acids. Further improvements such as food portion size estimations, features to send reminders to participants and the partial automatisation of coding food intake are under investigation (Table 2).

#### The Netherlands

Dr Evelien de Boer from the National Institute for Public Health and the Environment (RIVM) presented on the 2012–16 DNFCS which used two repeat non-consecutive 24 h recalls^(^^43^^)^. In the youngest (1 to 8 years) and the oldest (70 to 79 years) age groups, parents or participants were also asked to keep a food diary on the day preceding both 24 h recalls. The 24 h recalls were conducted by dietitians using the standardised computer-directed interview programme GloboDiet (IARC^©^), with responses entered directly into the computer^(^^5^^)^. Dr Boer highlighted that 24 h recalls coded through GloboDiet were linked to the national food composition^(^^44^^)^ and supplement databases^(^^45^^)^ which enhanced the estimation of habitual intake distribution through statistical modelling techniques. This level of detailed data was also useful for research questions on food safety, healthy diets, food sustainability and food policies. Furthermore, the dietary assessment method was in line with the guidelines of the European Food Safety Authority for the collection of harmonised food consumption data across Europe^(^^2^^)^. However, capturing foods consumed in a more efficient and accurate way continued to be a challenge in the DNFCS. Foods were not always identified well by respondents and sufficient detail might be missing. Moreover, due to the growing and changing food market, it was a challenge to identify the reported foods in a time-efficient way, in addition to the labour-intensive data processing (Table 2).

For the next DNFCS the RIVM was considering the use of advanced technology, for example, using barcodes during the data collection or the automated techniques for linking food consumption data with food composition data. Also, other data collection methods like mobile applications were being considered to improve data quality and accuracy. A potential option being explored was the use of a combination of two methods – a less intensive method (e.g. FFQ) covering large population groups which would provide rapid but less detailed data, accompanied by a detailed method (e.g. 24 h dietary recall) which would cover smaller population groups.

#### The UK

Dr Birdem Amoutzopoulos from Medical Research Council (MRC) Human Nutrition Research (now called MRC Elsie Widdowson Laboratory) reported on approaches used in the UK for the NDNS. The NDNS has used a paper-based 4-d estimated diary since 2008^(^^46^^)^. This traditional method had certain advantages including opportunity for self-completion by participants in their own time, supported by interviewer assistance, and sought collection by participants of food packaging and recipe information, thus enabling detailed dietary data collection^(^^20^^)^. Diaries were manually coded using the Diet In Nutrients Out (DINO) system^(^^47^^)^. However, whilst this approach aimed to ensure the overall accuracy of the dietary data, there were concerns about respondent burden, which might affect response rates, and data quality and therefore the degree of misreporting (mean energy intake:total energy expenditure was 0·73 in combined age/sex groups^(^^46^^)^). The time taken to manually code the diaries led to a time gap between data collection and coding which could mean that missing foods detailed as seasonal foods were no longer available. This method also has considerable cost implications and constraints on the timeliness of delivery of results data. There has therefore been interest in the potential to use new technologies to assist dietary assessment to overcome some of these challenges (Table 2). In 2014, the UK Department of Health commissioned a literature review of new technologies with relevance to the NDNS^(^^48^^)^. It concluded that current evidence was insufficient to either identify an appropriate tool or support a recommendation to fully implement new technologies in the NDNS at that time. Furthermore, although it was perceived that the main advantages of new technologies were likely to be cost saving in relation to data processing, or improving compliance, the review highlighted a lack of evidence demonstrating these advantages in practice. However, the review strongly recommended filling the evidence gap by investment in good-quality feasibility, cost-effectiveness and validation studies^(^^48^^)^.

Various UK research has developed and included use of new technologies in dietary assessment generally in settings focusing on specific groups of the population^(^^12^^,^^13^^,^^14^^)^. Recent examples are Oxford WebQ, a web-based method for assessment of previous 24 h dietary intakes tested in large-scale prospective studies^(^^12^^)^, myfood24^(^^13^^)^ and INTAKE24, an online 24 h dietary recall system^(^^14^^)^. Myfood24 has been validated in adults compared with a suite of biomarkers^(^^49^^)^ and relative validity has been demonstrated in adolescents^(^^50^^)^ and INTAKE24 was tested in the Scottish Health Survey^(^^51^^)^.

In 2017, the third wave for NDNS RP (2018–22) was commissioned, encompassing plans to consider alternative dietary assessment approaches more aligned with recent technological developments in the field, and which may provide greater opportunity for automated data collection, greater cost efficiency, reducing misreporting and maximising participant response.

#### The USA

The USA NHANES uses the computerised 24 h recall data capture via the US Department of Agriculture's AMPM^(^^3^^)^, now also used in the AHS^(^^35^^)^. Subar *et al.*^(^^9^^)^ have developed an Automated Self-Administered 24-hour Recall (ASA24) based upon the AMPM which could be used by participants in large-scale epidemiological studies. A large study indicated a reasonable level of comparability between the ASA24 and AMPM for nutrient and food intake^(^^52^^)^.

Like the ASA24, many of the new technology developments highlighted for potential application in population surveys have been web-based applications. In contrast, at the ICDAM9 Panel Dr Carol Boushey (University of Hawaii Cancer Center) presented information on a smartphone application (mobile food record, mFR) used to capture images of food intake^(^^15^^)^. With the mFR, individuals capture images of each eating occasion which are then sent automatically to a central server for processing^(^^53^^)^. A system referred to as Technology Assisted Dietary Assessment (TADA) that can be embedded for use with the mFR includes instructions on how to take a good picture in an effort to increase data quality whilst minimising the need for staff assistance^(^^54^^,^^55^^)^. A study^(^^56^^)^ tested the mFR using doubly labelled water and showed that the mean percentage of under-reporting was between 10 and 12 % for adults. This suggested the accuracy of the mFR to be comparable with traditional dietary records^(^^56^^)^. Besides, the mFR was well received and its usability was rated as easy in the studies in which it has been used^(^^57^^,^^58^^)^, which were similar in size to the evaluation studies of web-based dietary assessment methods^(^^12^^,^^14^^,^^51^^)^.

Progress being made with mobile technologies such as the mFR would suggest that these methods would soon be ready for larger-scale studies^(^^59^^)^. The increasing use of smartphone applications might offer considerable opportunities for future nutrition surveys in both children and adults.

## Discussion

New dietary assessment technologies offer potential benefits in terms of cost and researcher and respondent burden, and therefore scalability of population nutrition surveys as well as the ability to produce dietary datasets more rapidly. As highlighted above, there has been a growing portfolio of research demonstrating their effectiveness and potential; however, their use has yet to be exploited fully within large-scale population surveys.

The following is based on a summary of the Panel and audience discussions chaired by Professor Janet Cade (University of Leeds). The discussions were centred around questions focusing on the subsequent progress in the field.

### Should we pursue the use of new technologies to measure dietary intake in national nutrition surveys?

Many developed countries fund national nutrition surveys on a regular basis, to provide governments with a reliable source of detailed quantitative information on food consumption, nutrient intake and sources of nutrients. These data are used to monitor diet and nutritional intake at a population level in order to provide the evidence base for developing and evaluating health policy and, where required, specific nutritional interventions. In various cases, the data have been also used in dietary exposure assessment of chemicals in foods. As such it is critical that such surveys deliver data which are population-representative and of the highest quality; considerable attention must therefore be given to the selection and application of suitable methods. Additionally, given the time-series nature of these assessments, population surveillance programmes also need to evolve and incorporate new methods as they emerge.

Surveys require effective strategies to maximise response rates and to minimise non-response bias; there was evidence that over the long term it was proving more challenging to maintain the high response rates that have been achieved in the past^(^^29^^,^^30^^)^. Variables that might negatively affect response rates are therefore an important consideration^(^^29^^)^. The increased emphasis and incorporation of digital technologies in everyday life have prompted researchers to consider whether the lack of new technologies available in nutrition surveys was having a negative impact on response rates as traditional dietary assessment methods become less acceptable^(^^48^^)^. In the USA, a comparison study showed that 70 % of Internet users (*n* 1081) preferred the ASA24 over the AMPM^(^^52^^)^. These results suggest that dietary methods incorporating technology might encourage users to take part in nutrition surveys. The number of smartphone users in the USA was 224·3 million in 2017 (69 % of population) and estimated to reach 270·66 million by 2022, with the number of smartphone users worldwide forecast to exceed 2 billion users by that time^(^^60^^)^. However, despite increasing ownership, mobile phone and Internet access are not ubiquitous and there were legitimate concerns that acceptability of new technologies might be low among some population groups (even those with access), mainly for those who were not proficient or familiar with technology. Previous NDNS participants, who were non-mobile device users, stated that they would not participate in a survey which did not provide a paper-based diary as an alternative to a technology-based approach^(^^48^^)^. However in this specific focus group opinions were divided and some smartphone users felt it was a disincentive not to have the option of using an application which they considered would be the most practical method given they carried their smartphones all the time^(^^48^^)^. In studies that assessed the feasibility of the ASA24, some older participants reported having smart phones or tablets which they were more comfortable using than laptops or desktop computers^(^^61^^)^. However, in field testing of myfood24^(^^13^^)^ and INTAKE24^(^^51^^)^, response rates were low among older people. Furthermore, completion of INTAKE24 (not interviewer led) was low among the overweight and obese individuals and those living in deprived areas, and only 34 % of study population completed at least one recall^(^^51^^)^. Based on the feedback received from respondents, the researchers suggested that additional reminders and face-to-face interviews encouraging initial participation could increase the response whilst still remaining cost-effective^(^^51^^)^, and these recommendations can be applied to other surveys planning to implement technologies.

Other valid reasons for pursuing opportunities generated by new technologies in national surveys included the potential for cost-effectiveness, reduction of time between collection and reporting of dietary data and improvement of data quality through reduced misreporting. The Panel noted that comprehensive research on the cost impacts of new approaches was still lacking and recognised that there was no information about costs associated with the development and implementation of such technologies in a survey setting^(^^48^^)^ and that these were likely to be different from costs and savings generated in a research setting. The Panel recognised that exploitation of technologies might provide efficient routes of access to participants, bringing about various benefits, including delivery of, or providing access to, the dietary data collection tool itself, enabling greater and more affordable geographical reach, delivering instructions for participants on how to use the tool, and issuing reminders to prompt timely or fuller data entries, and collecting digital images to identify missing foods or to help to precise portions consumed^(^^62^^)^. However, it could not be presumed that this alone would be sufficient to secure engagement and effective participation without appropriate protocols to place the tool effectively and secure full participant engagement in the field (see below).

The ability to improve data quality, including the potential to reduce misreporting, was likely to be an important driver for change. The application of technologies was considered potentially useful to improve the completeness and accuracy of data collected; the development of tools such as Technology Assisted Dietary Assessment (TADA)^(^^55^^)^ was particularly focused on this aspect. However, it was not anticipated at this time that technologies could fully eliminate misreporting given the multi-factorial complexity of this issue^(^^63^^)^. In a covert observational study, respondents completing the ASA24 reported 80 % of items truly consumed compared with 83 % in the AMPM^(^^62^^)^. The researchers leading the development of the ASA24 made a valid point that opportunities and relevance to research might come from the substantial cost savings offered by new tools in the context of comparable data quality^(^^64^^)^. In the validation study, myfood24 provided the potential to collect dietary data of comparable quality with that of an interviewer-administered 24 h multiple-pass recall^(^^50^^)^. Furthermore, comparison of INTAKE24 with interviewer-led 24 h recall in 11- to 24-year-olds also showed good agreement for nutrients^(^^65^^)^. As illustrated, a number of new tools showed promise in comparison with traditional methods in terms of data quality^(^^50^^,^^64^^,^^65^^)^; however, thus far, none appeared to demonstrate significant improvements in accuracy. In the national survey setting, there would be concerns that new technologies do not increase levels of misreporting, and it would be important to quantify changes in data quality to understand the impact of changing to a technology-based method for data continuity. However, overall data quality is dependent on many factors and the success of national surveys is not just in the accuracy and precision of individual data collected, but equally in whether the resulting dataset is of sufficient sample size and representative. For these reasons, like in many areas of research, the most effective protocols are frequently the product of a trade-off between different quality parameters and constraints including logistics and cost.

### What strategies are going to be most effective to incorporate new technologies to measure dietary intake in nutrition surveys?

Traditional dietary assessment methods might need to remain as an option in surveys, at least for some time, to ensure that all population groups are represented in the sample. Multi-modal approaches offering a choice of traditional and technology-based tools could provide an effective strategy. In this case, method harmonisation and validation studies would be required to ensure that dietary data collected via different methods can be brought together in such a way that they are compatible and comparable, and to facilitate longer-term transition. Another possible approach is the use of personalised dietary assessment methods based on respondent characteristics (e.g. educational status, physiological status, geographical location, technology use). Studies also suggested that some features of mobile technology (e.g. receiving visual messages or capturing images of foods consumed) might help to improve response and accuracy among key age groups and smartphone users^(^^58^^,^^66^^,^^67^^)^ whereas population groups with variable cognitive skills and computer literacy (e.g. young children, older adults and non-technology users) could perhaps benefit more from interviewer input, either face-to-face or telephone support^(^^51^^,^^61^^)^. The Panel stressed the importance of carefully considering the implementation of new technologies into nutrition surveys and the potential need for tailored support for study participants.

As noted above, whilst new methods may offer opportunities for nutritional surveillance, they nevertheless constitute a change of data collection method. Continuity of data is a critical issue for the monitoring of trends over time in order to reliably detect genuine changes in intakes. Statistical approaches and bridging studies might be necessary to enable comparability between methods over time and to evaluate the feasibility and reliability of implementing new approaches. Various statistical analysis and modelling techniques have become available over recent decades which could be used in nutrition surveys to address other issues related to methodology. For example, the Iowa State University^(^^68^^)^ and the National Cancer Institute^(^^69^^)^ methods are well-known techniques which can be used for estimating the habitual intakes of episodically consumed foods^(^^3^^,^^70^^)^. There are also statistical simulation models that could be applied to survey data to adjust for under-reporting error using external datasets on a similar population^(^^71^^)^ or by use of biomarkers^(^^72^^,^^73^^)^. NHANES is an example of a nutrition survey which has been using a combination of different dietary methods with the same completion protocol for all participants to lessen measurement error^(^^3^^)^.

### What should be the next steps?

The Panel discussion in 2015 concluded that there was an urgent need for multi-disciplinary research combining expertise in dietary assessment, food composition, behavioural sciences, public nutrition and technology. The research would benefit from the close collaboration of international experts in order to improve the current dietary assessment methods used in nutrition surveys and learn from each party's experience. Given nutrition surveys are commonly commissioned by government, there would also be need for strong stakeholder engagement with commissioners and policy makers, to enable confidence in new approaches, and to facilitate change through periods of transition. There is a lack of evidence and information about costs, pilot testing and validation of new technology in surveys^(^^61^^)^ and this evidence would be critical to ensure successful integration of new technologies into nutrition surveys. Since nutrition surveys incur considerable cost, the evaluation of cost implications of implementing new technologies is essential to support decision making through providing evidence-based information to survey funders. Equally, reliability, specificity and accuracy of new tools are important concerns^(^^48^^)^ which should be addressed in validation studies as well as using a representative and adequate sample size.

In any transition to new technology-based methods, there would need to be careful appraisal of their performance in the survey context. National surveys have gone to considerable lengths to sustain high response rates and to ensure the representativeness of data collected. It was the strong opinion of the Panel that robust assessment of the performance and impact of new technology-based tools in surveys should be conducted *in situ* to enable continuity of tried and tested survey protocols which were known to deliver the best outcome in relation to response.

## Conclusions

Traditional dietary assessment methods used in nutrition surveys are subject to a number of limitations including respondent and researcher burden. The development of new technologies in dietary assessment is an exciting and fast-developing field of research with the potential to address a number of the challenges that have long existed with traditional methods including respondent and researcher burden, response, data accuracy, efficiency of coding and data processing. Employing new technologies to facilitate dietary assessment has the potential to reduce the costs and time taken for data collection, coding and analysis, to improve participation and data quality. In the long term, implementation of new technologies could improve the response rates and reduce non-response bias as they might be viewed more favourably by future respondents. New technologies might also offer more opportunities to use multi-mode collection methods such as automated, self-administered FFQ and 24 h recall in surveillance. With the direction of increasing ownership and preference to use digital technologies in society and in everyday life, this shift is inevitable.

The 2015 ICDAM9 Panel Meeting concluded that nutrition surveys and population studies would strongly benefit from research studies exploring the implementation of new technologies with appropriate controls. Given the pace of innovation and development in both web-based and smartphone-based tools, the Panel also anticipated that it would not be long before such technologies were ready for application in larger-scale studies and population surveys. The Panel emphasised the need for ongoing research to address the validity, feasibility, reliability and cost-effectiveness of new technologies, including harmonisation with established methods internationally. Well-designed pilot studies and a multi-disciplinary approach will help address and overcome the challenges that might occur in this process.

Now, 2 years on, in 2018, we are at the cusp of change. The digital online age is making an impact more widely day by day on a global scale, the numbers of Internet users and computer/smartphone owners are increasing, with the acceptability and use of technologies in domestic, school, work and public life accelerating exponentially. The UK NDNS RP is considering the inclusion of dietary assessment methods more aligned with recent technological developments. In Scotland, the possibility of piloting Intake24 is being explored. In Sweden, a recent national dietary survey in adolescents (carried out in 2016–17) was completed using a web-based method, *RiksmatenFlex*. These examples show that developments in new technologies for dietary assessment in the context of national nutrition surveys are moving forward and should be closely monitored to evaluate their success and potential for ongoing enhancements.
